# Parental Decision-Making and Acceptance of Newborn Bloodspot Screening: An Exploratory Study

**DOI:** 10.1371/journal.pone.0079441

**Published:** 2013-11-12

**Authors:** Stuart G. Nicholls, Kevin W. Southern

**Affiliations:** 1 Department of Epidemiology and Community Medicine, University of Ottawa, Ottawa, Ontario, Canada; 2 Institute of Child Health, Alder Hey Children's Hospital, Liverpool, Merseyside, United Kingdom; 3 Department for Women's and Children's Health, University of Liverpool, Liverpool, Merseyside, United Kingdom; Edinburgh University, United Kingdom

## Abstract

**Objective:**

Newborn bloodspot screening is an internationally established public health measure. Despite this, there is a paucity of information relating to the decision-making process that parents go through when accepting newborn screening. This is important as screening panels are expanding; potentially leading to an increasing amount of complex information. This study sought to understand the factors that influence parental decisions and roles they play in the decision-making process.

**Patients and Methods:**

Qualitative thematic evaluation of semi structured interviews with parents whose children had recently undergone newborn screening in the Merseyside and Cheshire region of England, UK.

**Results:**

Eighteen interviews with first time parents (n = 12) and those with previous children (n = 6). Seven factors were identified as being either explicitly or implicitly related to parental decision-making: Experience, Attitudes to medicine, Information-seeking behaviour, Perceived knowledge, Attitudes to screening, and Perceived choice, all of which ultimately impact on Perceived decisional quality.

**Conclusions:**

These results indicate that while content is important, other contextual factors such as personal experience, perceived choice, and general attitudes toward medicine, are also highly influential. In particular, relationships with key healthcare professionals are central to information collection, attitudes toward screening, and the level of deliberation that is invested in decisions to accept newborn bloodspot screening.

## Introduction

Newborn bloodspot screening (herein newborn screening), in which a small amount of blood is drawn from a baby's heel and tested for a number of serious and life-limiting conditions, is one of the largest screening programs in the world. Today, newborn screening is implemented across most continents, with almost universal uptake. In the US alone, this equates to roughly 4 million infants per year [1]. Recent years have seen dramatic developments in technological capabilities. These advances have allowed programs to expand the number of conditions screened for at marginal extra cost, with some jurisdictions now screening for over 40 conditions [2].

In the UK, screening has tended to take a more cautious approach, with a limited panel of five diseases including PKU, cystic fibrosis, and MCADD. More recently pilot work has been undertaken to expand this to a total of ten conditions [3]. Moreover, in the UK, in contrast to the US but in-keeping with other European countries such as France [4], screening can only proceed on the authorization of parents on the basis of an informed decision [5]. In England and Wales verbal consent is sufficient, while in Scotland written consent is required [6]. Standard practice dictates that before proceeding to take the blood sample – usually collected by a midwife at the infants' home between the fifth and eight day after birth - health professionals, again primarily the midwife, are expected to provide written information in the form of the *Screening tests for you and your baby booklet* (http://www.screening.nhs.uk/annbpublications (Accessed 26th June 2012)) and discuss screening with the parents [6].

To date, research has tended to focus on parental knowledge [7], [8] or parental education [9], [10], with little research as to parental decision-making [11], [12]. These studies do not provide clear insight into parental decision-making or whether parents are making an informed decision; assessments of parental knowledge may not report on understanding or decision-making, but more simply reflect an individuals' memory [13]. Moreover, there is a lack of understanding with regard to the impact that recalled information has on the decision-making process [14]. Equally, while several studies indicate when and how parents receive information about newborn screening [15] and the content of this information [9], [16], these studies tell us little about how this information is used to inform decisions about whether or not to accept screening.

Studies from other contexts provide limited insight as determinants of uptake depend on the type of screening test [17]. Termination of pregnancy, for example, has been found to be a highly significant influence in the context of prenatal screening [18], yet is not a salient factor in the context of newborn screening. Indeed, a report produced by the UK Newborn Screening Programme Centre, which sought to summarise the data on communication with parents about newborn bloodspot screening, stated that:

“There is limited research reported about parents and professionals views and experiences of: pre-screening information (and none of antenatal information); consent for screening; the heel-prick itself and subsequent tests; the information provided with screening results; and in particular communication about carrier testing.” [11]

This study sought to address this deficit in order to better understand the factors that influence parental decisions and the ways in which they do this.

This was undertaken as part of a larger exploratory sequential mixed methods approach [19], in which the goal was to explore individual parent experiences of decision making, with a subsequent phase in which a proposed model of parental decision making was evaluated through statistical analysis. This approach was underpinned by a critical realist perspective [20] in which a retroductive logic is used to question the underlying processes or mechanisms that, if they existed, would lead to the phenomenon in question, i.e. the experiences related by the parents [21]. In taking such a realist ontology and accepting a relativist epistemology, we accept that knowledge is socially constructed but that there may be enduring mechanisms that exist to create patterns or regularities of experience that may point to causal factors. Put differently, from individual experiences we may posit mechanisms that could lead to such a phenomena existing. Moreover, this proposed mechanism may gain evidentiary support through the collection of quantitative data that is consistent with the proposed relationships.

This manuscript presents the findings of qualitative research investigating parental decision-making when accepting newborn bloodspot screening and reports key elements of the parent experience, positing relationships between these factors for future evaluation. In doing so, it is the first study to actively explore the decision-making process of parents of children who have undergone newborn bloodspot screening and identify factors that influence decisions to accept screening.

## Methods

Parents were recruited between December 2008 and May 2009 and were eligible for inclusion if they had given birth in the previous two years. Parents were excluded if their child was severely ill, had subsequently died, or if they were unable to converse freely in English.

Sampling was purposive in nature [22] and sought to engage with parents of differing socio-economic status, with location and associated area demographics used as a proxy in lieu of individual data. The study took place in the Merseyside and Cheshire region of England, UK. Whilst these conurbations are served by a single screening laboratory, population demographics vary widely as shown in Table 1.

**Table 1:** Characteristics of recruitment areas.

	Location			
Data profile item*	Macclesfield	Sefton	Wirral	Liverpool
Rank of the average Indices of Multiple Deprivation Super Output Area (Scale 1 (most deprived) to 354 (least deprived))(2004)	276	78	48	1
Annual average House Price: Overall (2005)	£238122	£156855	£140153	£115244
% of children that live in families that are income deprived	9.9%	23.7%	29.9%	44.9%

* Data taken from the audit commission website: http://www.areaprofiles.audit-commission.gov.uk. Accessed 9 October 2009.

Parents were identified through laboratory records held by the Merseyside and Cheshire Regional Screening laboratory, branches of the National Childbirth Trust (NCT) and local Sure Start schemes. Parents who declined screening were identified through laboratory records and over-sampled in comparison to actual occurrence. All babies whose parents had declined screening were selected for approach (n = 7).

In all cases, parents who met the inclusion criteria and who failed to meet the exclusion criteria were approached by letter in the first instance. This invitation letter introduced parents to the study, as well as providing contact details. Included with the invite letter was a patient information sheet which provided more details regarding the project. If parents wished to take part they indicated this on a reply slip which was returned in an included envelope. This reply slip asked for contact details so that an interview could be arranged. If no response was received within two weeks of sending the letter then a reminder letter was sent. If no response was received following this letter then no further action was taken.

Data collection was by semi-structured interviews [23] undertaken by one researcher (SN). Interviews took place in a location of the interviewee's choosing, which in most cases was their own home. Interviews were audio-recorded using a digital recorder, although in one instance field notes were taken at the request of the interviewee. All recorded interviews were transcribed and anonymised. Transcripts were imported into Atlas.ti Qualitative Data Analysis (QDA) software [24] to assist with management and coding. As a process of validation, transcripts were made available to interviewees for comment [22]. No further comments were received from participants.

Interview data was analysed in an inductive manner using a thematic analysis approach [25]. Thematic analysis shares many features with other methods of qualitative analysis such as Grounded Theory in so far as textual data is coded and labelled and, in an inductive approach, are grounded in the data. The paucity of prior research on newborn screening precluded the use of any pre-determined coding scheme.

As indicated above, the focus of the analysis was not only on the development of themes that would be taken into the second stage of the study but also the development of causal relationships between these. As such it diverges from traditional Grounded Theory approaches where the development of a general or substantive theory is a key goal. This process of coding was iterative with the codes being developed using the constant comparison method allowing for the revision, combination or separation of codes in light of new data [26]. Each newly coded incident was compared both within and across cases to previous incidents in order to refine or revise the code [27]. Following the initial coding of transcripts, codes were then grouped into overarching themes.

### Ethics statement

All parents provided written informed consent to take part and the study received formal ethical approval from the Liverpool (Adult) Research Ethics Committee (Reference: 09/H1005/66) and the Lancaster University Research Ethics Committee. The study was also submitted to the UK National Institute for Health Research Clinical Research Network (NIHR CRN) Portfolio (ID number: 8106).

## Results

All parents who declined screening (n = 7) also declined to take part in the interviews (n = 2) or failed to respond. We therefore report interviews with 18 parents who had accepted newborn screening. This represented an overall response rate of 36.7%, although this varied with a lower uptake of parents identified through laboratory records. Theoretical saturation was reached with no new themes emerging.

Interviews were with both primaparous - first time - parents (n = 12) and multiparous parents - those who had more than one child (n = 6).The majority of interviews were with mothers, although two interviews were conducted with fathers who were spouses of mothers who were interviewed. When both parents were recruited individual interviews were conducted. The greater recruitment with mothers is in keeping with much of the newborn screening literature [28]–[30], but also reflects current practice in which maternal details are recorded within the newborn screening system, a process which is based on the primacy given to mothers as guardians[31]. Only one parent had a child affected by one of the screened for conditions, although this was not their most recent child. Another mother had a child affected by an inherited metabolic condition with similar implications to Phenylketonuria. The interviews ranged in length between twenty and forty five minutes.

As hypothesised by the purposive sampling frame, participants varied in terms of their personal circumstances and experiences as well as age and number of children. However, specific data was not collected on these demographic characteristics and only spontaneously offered data is available. As the data comes from a small sample it would be inappropriate to apply statistical notation, such as percentages, when discussing the thematic analysis and the number of respondents who discussed particular themes. Whilst the intention was to interview parents individually, on occasion partners were present during part of the interview. In all instances additional consents were taken to include their input.

Seven key themes emerged from the interviews as being either explicitly or implicitly causally related to parental experiences regarding consent for newborn bloodspot screening. These themes are classified as Experience, Attitudes to medicine, Information-seeking behaviour, Perceived knowledge, Attitudes to screening, and Perceived choice, all of which ultimately impact on Perceived decisional quality.

### Information-seeking behaviour

Whilst the focus of this study is on parental decision-making to accept newborn bloodspot screening and the factors that affect this decision-making process, an important prerequisite is the information on which the decisions are based. This was also talked about at length by parents when they discussed their experiences of the heel prick. As reported previously [15], a range of information sources were used by parents including official National Health Service (NHS) leaflets, books, the internet, friends and family. The most consistent reference was made to the midwife who played a central role in information provision for parents. Parents cited difficulty in finding the time to read written information during the post-natal period and the advantage of the midwife was that the visits provided an opportunity to gather information quickly during time that was already set aside.

The lack of use of written materials was sometimes compounded by the way it was provided with other commercial literature or left for parents to find. In contrast to other research [32], the internet was not a great primary source of information for parents. The decision to accept the heel prick was straightforward and the internet served a need only when researching a contentious issue, such as vaccinations or pre-natal testing. In the context of the heel prick, the internet was largely used as a supplementary tool for gathering information not for making a decision. One acknowledged limitation to the use of the internet was a lack of quality control. One mother, who sought information after not receiving any from the health service, recalled:

“I did look on the internet […] but you don't know, especially with the internet, you don't know what you should […] take and not take, because it could be very scary if you looked at that, you know what things, the statistics and everything. You could get quite scared if you looked on like one internet site, you know if you googled it and put it in.” *M#10, primaparous mother*

### Experience

Personal experience had a significant role in the information-seeking behavior, particularly for multiparous parents. For some this effect was to reduce the amount of information they sought, instead relying on their own experiences. Yet for others prior experience of ‘the system’ and processes of childbirth meant that they were able to focus on the specifics of the information:

“I: So what's changed this time?

M#17: But this time, I think you change as a parent anyway and you gain in confidence, so.

I: Why do you think that is, why do you think you've sort of, where do you think you've got your confidence from?

M#17: [laughs] Well it's your child and you want to protect them at the end of the day and make sure that everything's right for them, you know, and it's learning that your views do count and learning that you are the expert on your child, because at first I thought ‘oh my God I don't know what I'm doing’, you know, it was like shock horror and then its, it's learning.” *M#17, primaparous mother*

This experience meant that parents were able to not only question medical authority, but also to have pre-emptively thought about situations that they would face and prepare for these. This experiential knowledge encompassed both embodied knowledge, that is subjective knowledge that comes from one's own personal experience, but also empathic knowledge which is subjective knowledge gained through the interaction and association with others, such as friends or family [33], [34].

In contrast to the way the midwife and the written resources were used, friends and family were rarely used to gather technical information. Instead, they were used to prepare for the screening process; how the baby cried when blood was drawn and the emotions that they felt, rather than any detail on the tests. As one mother recalled:

“Well, erm, I kind of knew about it through friends and my sister in law, […] and I remember there just being this sort of dreaded day three, midwife comes to the house and, and you just want to punch them. Was the general feeling I got from these other mums because they're making your baby cry kind of thing.” *M#5, primaparous mother*

As such, experience was a valid source of information. The use of experience as a valid source of information was not restricted to friends or family. In some instances parents appeared to be using experience, assessed through a proxy of age or number of years in a particular field, as a way of validating the information that they had received and so determine its legitimacy.

“I think they're [parents] probably as important, because I'm quite close to my parents and, and obviously being that close to somebody who has had children, mum was, mum was very useful, and also my husband's mum er was very useful as well, cos she's a nursery nurse and has loads of experience.” *M#12, primaparous mother*

Hence the experience of her mother-in-law, within her role, was a method of validation for the information she provided. Trust was placed in the individual, and this individual trust was generated not only by regulatory identifiers, such as qualification or association to an institution that implied both competence and worthiness, but also through experience and interaction. This was explicitly stated by one mother who had cited the midwife as an information source:

“I: Erm, is there anything about the fact that it was sort of the midwife that's good, other than the fact that she works at the hospital, is there any other benefits that you can think of?

M#10: Well I think because she's know, with a baby, she's more, she's a bit more experienced isn't she.” *M#10, primaparous mother*

Thus the experience of the midwife was linked to competency and trust and as such an additional tool of validation alongside her qualifications.

#### Assumed knowledge with experience

Whilst parents saw positive aspects to their experience in assisting them to make their decisions, it was also indicated that when experience, and particularly knowledge gathered through experience, was assumed, this could lead to them being less informed. In part this was due to professionals assuming that the parent would know about the heel prick:

“[…] I actually don't remember being told the details as much that time [with 2nd child], now whether that's because they said do you know what the heel prick test is, to which I've gone yes, because obviously they've done it before so they've just thought OK, take the blood and send it off. That's possible, that once you've had one child a lot of details are then skimmed over, that they just assume. I, and I probably assume, ‘oh yes I know' when actually, really, if you'd have dug a bit deeper I actually didn't really know what it really was.” *M#16, multiparous mother*

This is reflected in the way that parents often had greater recollections of their first child having their heel prick than their second. The assumption within recruitment was that the heel prick would be fresh and clear in their mind. Here the suggestion may be that even when parents have a second or third child, it is the experiences of their first that may be more lucid. Consent was experienced as less thorough for their second child where it was felt that the professional assumed that they knew about the screening, resulting in potentially less thorough information provision.

#### Experience and decision-making

For some parents the decision to accept screening was largely based on their previous experiences with the heel prick. For those multiparous parents the decision appeared straightforward:

“She went through it very briefly with me and explained about the different reasons for doing the test and I'd had the test done on [son] and we'd already made that decision with him so to make the same decision with her was much quicker and easier like yeah, yeah, whatever come on, come round and do it […]” *M#7, multiparous mother*

And later:

“But it was much easier for me to decide for her to have it done than with [son] and we went through quite a lot of erm, […] grief and research and soul searching with [son] the first time about immunisations and testing and amnio, all those sorts of things but when it's second time round you've already been through that decision making process, so it's like are we gonna do what we did the first time or have we changed our mind here? So in a lot of ways it was easier.” *M#7, multiparous mother*

This perspective, of reviewing the experiences of their first child and basing their decision on that experience, was replicated in other parents. Thus, whilst experiential knowledge from friends, family or professionals was an important information source, when this experience was personal there appeared to be far less deliberation or conflict. As suggested, this may be reflected in the ability to recall more clearly the heel prick from their first child. The caveat here is that these parents had not previously had an adverse outcome to their decision, or a decision which they had regretted. Had this been the case, it may have been that personal experience would have had a different effect on decision-making.

### Attitudes towards medicine

For some mothers the decision to accept screening was informed through more general attitudes towards medicine. These attitudes were often affected by their own experiences of the healthcare system and their perceptions of how the healthcare system in the UK works. In particular this was linked to a perceived knowledge of the processes through which testing becomes available. This was occasionally framed by the financial constraints within which the NHS operates:

“M#11: Yeah, yeah. They obviously know what they're doing, they're trained professionals and they wouldn't just rou… you know cost wise and things like that, do these things if it wasn't necessary. You know, knowing the way the NHS is and things like that, I couldn't see them doing it just for something to do.

I: Yeah. Sorry, what do you mean, you said knowing the way the NHS is?

M#11: Doesn't, with funds […] you know, they all seem to be short of money, you know when they, you know, you want these cancer drugs and that but they say they can't afford it and things like that. So I shouldn't imagine something like that [newborn screening] wouldn’t be carried out unless it was absolutely necessary.” *M#11, primaparous mother*

Without explicitly mentioning Herceptin or similar medicines, which have received press coverage because of the variation in provision [35], this parent has neatly summed up the perception of the approval process. This perhaps clarifies the behaviour of parents who accepted screening without seeking much information. By virtue of screening being offered by the NHS, it is perceived to have been reviewed and assessed to a level whereby the NHS is happy to provide it, which indicates to parents that this is a good thing to have.

Decisions were also informed by personal contact with healthcare professionals, and particularly the midwife. Hence, while the midwife was an important source of information she also played a significant role in parents accepting newborn bloodspot screening. For some parents, as was the case with information provision, the offer by the midwife suggests a level of quality and it was trust in the midwife who was offering the test that led them to accept.

Trust in the midwife was built through their presumed qualifications - a presumption based on their affiliation to hospitals and the NHS more generally - together with parents' expectations of training. Ability was demonstrated through knowledge, itself assessed by answering questions or providing information. This was combined with personal qualities to generate trust. These personal qualities were demonstrated through the interaction with parents:

“I: And do you think, maybe, if she couldn't have answered your questions there and then it would have been a bit…

M#8: Yeah, yeah, well, it depends. I mean obviously if I would have asked her a question that's not, is something that she'd need to research, if she would have said I need to research that, then that would have been fine.” *M#8, primaparous mother*

So a level of personal reflexivity and honesty was deemed to be important, even if that was to the detriment of the immediate level of information she could provide.

Whilst trust in the midwife and the NHS was relatively high, parents were less trusting of administrative aspects of hospitals. In particular, concerns were raised over the provision of results. Parents recalled being told that ‘no news is good news’ and that they would only receive results if there was anything of concern. Hence attitudes towards midwives and the NHS more generally, interact with specific attitudes towards screening and information-seeking behaviour; both of which are important in parental decision-making.

### Perceived knowledge and understanding

Parental knowledge was not assessed through direct questioning. Given the shortcomings of knowledge assessed by recall [36], parents were asked to relate their own perceptions of their knowledge. When talking about their experiences of the heel prick, most parents were self-deprecating; with a number openly stating that they could not remember anything. In particular parents sometimes felt they had a poor technical knowledge, unable to recall details such as the exact names of the conditions, the specific prevalence or similar. To this end several mothers suggested that severe tiredness was detrimental to their ability to remember details. As one mother explained:

“But when you've just got a new baby you, you're knackered aren't ya, and you're looking after the baby and just cos I'm, I couldn't, I couldn't have told you the next day and, you just forget.” *M#18, multiparous mother*

Others talked about how physiological or psychological changes, such as hormones and emotions, affected their memory. It may well be that parents of unaffected children have a decreased knowledge, possibly due a perceived lack of need to remember details. Parents of children who are affected by the screened for conditions may have a greater knowledge, particularly for the condition which affects their child.

Despite a self-professed lack of technical knowledge, when parents began to talk about the heel prick, many exhibited some knowledge about the screening. This knowledge was sometimes presented in their understanding of why the heel prick was taken. This occasionally included what may be viewed as technical information about the causal pathway of the conditions:

“I can't remember if it's this one, is it to do with finding out certain, if, if ya child can only have certain food, foods and stuff like that, yeah. That's the only thing I can really remember really. And obviously if they, if they find out at this early stage then it can, then it can erm be very beneficial and it can basically, you know, eradicate any sort of future problems, can't it, and things like that.” *M#9, multiparous mother*

The indication here is that whilst memory may be compromised, the parents understanding of implications are not.

### Attitudes towards screening

As already detailed, general attitudes towards the NHS and the midwife appeared to dominate attitudes towards newborn bloodspot screening. Whilst potential concerns were raised, these were all perceived to be low risk. The main concern voiced by parents was the potential pain that would be inflicted upon their child. This was often revealed when parents were asked if there would be anything that would stop them having their child screened. The focus on the potential for distress and the effect of the disease may reveal why parental knowledge of this and procedural aspects were greater than other, technical, aspects. Despite these concerns, the actual risk of distress was seen to be minimal:

“Erm, yeah, but it's something that you know's not gonna…and it's short term it's, you know, and they're not going to be psychologically marred by it for the rest of their lives, erm, and you can comfort them.” *M#4, multiparous mother*

This perception was strengthened when they were provided with information as to how they could take steps to minimise the distress to their child. One particular factor that affected the perceived level of distress was parental opinions on the cognitive abilities of the child. The benefit was that at such a young age the child would not remember it and so not be adversely affected.

#### Perceived risk: better out than in

Some parents spontaneously compared the heel prick with the measles, mumps and rubella (MMR) vaccinations which are offered to children. This distinction was based on experience with older children, but also on issues of risk and safety:

“[…] also I think because there's a chemical element to the MMR and all the vaccines you go for, whereas the heel prick's very straightforward, erm, you know, it's literally just taking the blood isn't it and testing that blood […] externally, so, erm, it's not, you know, if you're not filling your child with something that you don't necessarily think that would, that they would have anyway, that's an unnatural element, then I think that's more questionable than, than just kind of like a small scratch […]” *M#12, primaparous mother*

Accordingly, the heel prick was a minimal risk intervention. In line with their own expressions of knowledge and understanding, the procedural aspects appeared to weigh most on their minds in terms of any potential harm or benefit that may result.

Whilst risk was weighed in terms of harm from the process, benefits were weighed on the basis of the diseases being treatable. This was conveyed when parents were asked about the possibility of screening for non-treatable conditions. Whilst treatability has been reported as an important concept [37], this was found to be a subcategory of what we call the ability to act; that is parents could do something with the results from the screening:

“[…] in a way you see with a result from a test like that you're knowing something that you're going to know anyway about baby eventually, so you want to know earlier and it's better to know very early….the process […] You would have found out anyway eventually erm […]” *M#15, primaparous mother*

Testing was supported in terms of knowledge being gathered earlier, allowing treatment to progress. This knowledge was also beneficial as it was felt to help parents cope, something that and has been invoked by some commentators as a reason for expanding newborn screening [38]. In the current context it may also be seen in terms of adopting treatment strategies that would allow them and their child to deal with the disease.

### Choice

When discussing the heel prick with parents it soon became apparent that the initial conceptualisation of parents conscientiously considering information, making a decision based on this information and then enacting this decision, was not only overly simplistic but in some cases erroneous.

The perception of newborn screening as routine was perpetuated by its inclusion with other post-natal checks, checks that were part of standard post-natal care and which led some to the conclusion that the process was an automatic one [39]. This normalisation can be seen in the way parents talk of the processional nature of screening:

“It was just, as I said, it was just one of those things that was all part of that, all of this, this big machine that happens as soon as you, as soon as you have a baby. You know, things like the health, triggering all these visits from people…it’s just, not like a tread mill but you realize that you are part of this as I said, system.” *M#3*, *primaparous mother*

#### Dualistic representations: importance and insignificance

Parents talked about the heel prick being presented in a way that sought to maximise uptake and minimise concern. As already suggested, the presentation of screening as routine served to suggest it was insignificant. This was compounded by the way that midwives talked and were dismissive about susceptibility and likelihood of a positive-screen. Yet this was counterbalanced with the importance placed on the heel prick by the midwife, with parents not only interpreting it as important, but also explicitly recalling being told this.

“If anything because of the way she brought, […] the way she explained it, and what it was for and stuff, and it made ya think oh yeah, and obviously it must be important that it needs, you know having, needs to be done.” *M#9, multiparous mother*

This perception was enforced by the fact that the midwife actively recommended the screening. As a consequence one can see how the perceived routine nature of screening; that it is offered to everyone in a way that does not draw attention to it, together with a firm recommendation that one should have the test, contrives to suggest to parents that the test should be taken.

#### Perceived choice

Parents recalled being told that “we're going to come and do this…” and so, as reported in other studies such as those by Parsons et al.[40], screening was seen as a fait accompli. Indeed several parents prefaced their interviews by saying that they hadn't considered the screening a choice.

For some, the timing of the heel prick meant that actually making a considered and informed choice was difficult, if not impossible. This difficulty was clearly articulated by one mother who had only received her information post-natally:

“[…] I don't think you're given any time cos your just told that they're going to do it and they need to do it, […] They don't say we'll leave it with you to think about and read, the literally say, blah-blah-blah the test and right here's the needle and they're about to take the blood. So it's a very, very quick process and you're not given any option to think about it.” *M#16, multiparous mother*

For others the difficulty lay in other aspects of the time, with some questioning their ability to focus and make a decision as they would ordinarily:

“But if, if they were gonna do a heel prick test tomorrow […] On [daughter] I think I would erm, yeah my er, my, my thoughts about it might be different I suppose than to one day after giving birth to her […] If you asked me at the time I'd probably think oh yeah, well don't be so ridiculous, of course I'm coherent of course I can, you know, you think you're pretty amazing once you err, the day after you've given birth actually [laughs], yeah, yeah, you're pretty infallible really so err, dunno.” *M#6, primaparous mother*

The suggestion being, that the timing of newborn screening information, which for a number of parents in this study took place after the birth of the child, was potentially detrimental to them giving of an informed consent.

### Decision-making and decisional quality

For some the decision to have the screening had been made prior to the provision of information. Despite having made their decision, these parents wanted to know about the implications of the decision. In explaining this, one mother suggested that there is a perceived need for testing that doesn't necessarily have to be informed prior to making the decision:

“I: Yeah. Was that, did you speak to her at the same time or was that before or after.

M#15: Same time, around the same time so a mixture of all of them – it wasn't gonna change my decision though.

I: No, you'd made your decision.

M#15: Yeah, but I wanted to know what I was letting myself in for.

I: OK, yeah, it was more about preparation rather than making your decision.

M#15: Yeah, yeah, yes definitely.

I: Right, so with the […] so with the erm, decision you said it was sort of, you wanted to make sure everything was all right, was there anything that stood out that ‘oh this is an important thing because x' that made you say yes - was there anything in particular?

M#15: No 'cos I'd made my mind up before that before I knew about the information.” *M#15, primaparous mother*

The decision to accept the screening appears to have already been made by this mother and on the basis of a predetermined principle. In exploring this the mother states that:

“I think it's just because of the person I am I think I hadn't wanted to refuse any tests for [daughter]. I do for me because I, I'm making the decision for myself, but for [daughter] and I think I had to consider my partner as well […] I knew what he would say anyway as he would want all the tests erm, but erm, I wanted to find out what the thinking was current thinking about it.” *M#15, primaparous mother*

Thus the process of active information gathering for this parent was not to inform the decision; the decision has already been made to accept the screening, but to make that decision an informed one. The driver behind the decision is a predetermined principle, beneficence, invoked as a reason for screening on the basis that treatment was available. This was implicitly tied to attitudes towards medicine in general. A positive attitude towards medicine, together with a general attitude to act to maximise benefit, saw this parent accept the screening on principle, and then inform themselves by gathering information.

This support of beneficence appeared in contrast competing principles invoked earlier in the pregnancy:

“I know it's really weird, but the sixteen week test I don't, wouldn't wanna know because I'm having the baby anyway […] But when she's born, I'd rather know if there was anything wrong with her to try and treat her.” *M#17, primaparous mother*

So again, post-natally there is a focus on acting on information and actively-seeking testing. This is a change from pregnancy when the principle of nonmaleficence; that is the norm of avoiding the causation of harm [13], appears to be invoked so that they do not cause harm to the child through the application of potentially risky testing. Once the child is born, a level of risk, or even harm, is to be tolerated so long as the perceived benefits outweigh these risks or harms. Yet beneficence was not all conquering, and the specific context may mean that detailed information about the process can still be important. In describing her encounters with clinicians regarding the provision of vitamin K one mother noted that:

“[…] when we were in hospital the first time we said we didn't want him to have the K, vitamin K injection […] And erm, there was this big gasp of you know, well nobody says they don't want it […] It was really, really strange so I thought well it’s a bit odd really and they just thought well why don't you want it, I said well I don't want him to have injections and there is an alternative and they can have it orally […]”*M#7, multiparous mother*

Hence, for this parent, the availability of alternative modes of delivery meant that the principle of nonmaleficence was again invoked, highlighting the complexity of reasoning. Whilst there is a low perception of distress and the lack of alternatives it may be that the principle of beneficence is of greater consequence. With a greater distress:benefit ratio and alternative forms of administration then the principle of nonmaleficence becomes more prominent until a tipping point is reached whereupon parents are no longer willing to proceed. All of which suggests that there is a complex process of checks and balances covering not only the calculation of risk estimates in terms of likelihood, but also size of effect and potential alternatives as well as underlying principles that parents assess when considering the heel prick.

## Discussion

A number of studies have explored parental knowledge of newborn screening [8], [41], [42] and attitudes towards expansion [43], yet few have considered the decision making process that parents go through when choosing to accept screening. Our study sheds light on these processes indicating that the way screening is presented and offered may be as important as the immediate information provided.

The findings must be considered in light of the limitations of the study. The purposeful sample of interviews, while selected to represent a mixture of geographic locations with associated socio-demographic levels, was not a random population sample. Further limitations are the small sample size and the fact that the small number of parents who declined newborn screening were not willing to take part. Research with parents who declined screening would add an important insight into this process, but engaging with these families is a tremendous challenge.

Whilst risk:benefit analyses were present within the discourses of parents recalling their decision-making, this tended to be limited to hypothetical scenarios of imagined barriers to screening. The question of risk was rarely discussed beyond these hypothetical situations, with the potential benefits being at the forefront of parents’ minds. A key motivator for accepting screening was to act on the information provided in order to benefit the health of the child. This is consistent with a number of other studies [44]–[46], and reflects existing screening criteria [47] and much of the discussion around the expansion of newborn screening panels [37], [48].

However, our study also identified a number of contextual factors that served to inform parental decisions and which have received little attention in the newborn screening literature. In particular we identified attitudes towards the healthcare system, and prior experience of newborn screening as important factors when parents are deciding to accept newborn screening. Additionally, we note the important role played by the relationship with key healthcare providers (here the midwife). While presented as discrete factors, these are highly interwoven with prior experiences and relationships with healthcare providers informing information seeking and perceived knowledge. Conceptually, these findings are consistent with the theory of planned behavior (TPB) [49]. Moreover, they show concordance with previous definitions of informed choice based on the TPB and in which individuals are deemed to have made an informed choice if they have availed themselves of relevant information and made a choice in accordance with their values and attitudes and then implemented this choice [50], [51].

As per the TPB, parents indicated that decisions were influenced by attitudes toward screening, subjective norms in the form of expectations of compliance, but also perceived behavioural control in terms of the perceived ease or difficulty of performing the behavior. The latter is clearly illustrated in the way in which multiparous parents talked about the ease of making screening decisions regarding second or subsequent children. However, while studies of informed choice have suggested that attitudes and knowledge are independent categories [52], our results suggest that the relationship between knowledge and attitudes may be mediated by experience – both personal experience of screening but also embodied knowledge of the healthcare system. Consequently, we may parse out the TPB factor of perceived behavioural control into its constituent elements of perceived availability of choice, ability to make a choice, and experience in order to further examine the interrelationships between these sub-elements.

Experience of the healthcare system was an important factor in contextualizing the provision of the screening program and the formation of trust, but when there was prior personal experience of screening, this played a dominant role in decision-making suggesting that parents may be substituting experiential knowledge for clinical information provided by information materials or the midwife. This is borne out by previous UK studies in which Smith et al., found that parental awareness of screening was higher in mothers with multiple children compared to first time mothers [41], although both groups showed a poor level of awareness. In a later study, established parents were found to be no more knowledgeable of the screened for conditions than were first time parents [53]. Our interviews support these findings and suggest that practitioners should be careful not to assume knowledge in mothers who have already had children go through the screening process. Information provision should be consistent and comprehensive irrespective of parity.

Our data suggest that a potential reason for this, and the greater recollection of experiences with first born children, is that compared to first children, the decision making process for subsequent children is less deliberated. Having already made the decision for their first child, the process of deciding for subsequent children is expedited on the basis of prior personal experience, potentially leading to the lesser recall of information in later children. This mediating role of experience is congruent with research into decision-making for prenatal testing and in which personal experience was a significant factor in decisions relating to prenatal testing [18], [34]. This indication that parental decision making is not principally informed by the immediate information provided by information leaflets or program website is important given the extensive research into the development of patient information materials for newborn screening over recent years.

The relationship with the midwife appeared to play a substantial role not only in terms of information provision but also in terms of reducing the burden of decision making. Indeed, the discussions regarding the receipt of information from the midwife can be seen as an example of what Case has referred to as ‘passive searching’ occasioned by parental attendance at antenatal appointments and scheduled visits by the midwife [54]. This passive receipt of information – as opposed to an approach of actively seeking out information about newborn screening - is consistent with Wilson's model of information-seeking in which non-active modes of searching and acquisition take place [55] and was reported to facilitate information uptake by overcoming barriers to information acquisition, such as the poor way in which leaflets were described as being provided. This underlines the centrality of the midwife as a source of information for parents about newborn bloodspot screening.

However, the parent-midwife relationship was also prominent in the actual decision making process, as has been noted in previous studies [40], [56], [57]. In each, trust in the midwife or healthcare professionals was an important factor, even overriding the need for information in some parents [40]. The present study confirms this important role of the midwife, but also offers an insight into the causal determinants of this trust. Trust was generated through both experience with the midwife, but also the wider health service. For some mothers the perception of the screening as routine, contextualized by knowledge of the ethical processes and financial limitations of the health service, was seen to verify its acceptability. This appears not to be a systematic processing of the immediate information about screening, yet is not a simple heuristic such as ‘experts can be trusted’ [58]. Similar findings have been noted in the context of prenatal screening decisions, and where trust in the healthcare system was found to lead to the perception that screening was a helpful technology [59], [60]. Consequently, individual trust in healthcare professionals and system level trust appear to be mutually reinforcing factors that influence parental decisions to accept screening; health professionals are given a ‘warrant for trust’ by being associated with a health profession yet at the same time the institutional trust is established through interpersonal interactions [61].

The caveat here is that, in the main, the parents who took part in our study did not have a child affected by a screened for condition and so we cannot assume that this finding is generalizable to parents whose first child had a screen positive result. Furthermore, the lack of parents who declined screening means that we are unable to comment on the decision making of this small but important group of parents.

In conclusion, our results are consistent with prior conceptual work regarding informed choice and the important roles played by knowledge, values and attitudes when making decisions regarding population screening. However, our results indicate that while *content*, in terms of the information provided to parents about the risks and benefits of screening, is important, other *contextual* factors are also highly influential and play important roles in mediating the relationship between knowledge and attitudes. In particular we note the important role of experience.

As newborn screening programs expand and become more complex with, for example, some conditions being tested for by DNA analysis, it is increasingly important that parents are supported in their decision-making process. Parents who consent to newborn screening should do so with confidence that they understand the procedure and implications. Our research suggests that the interactions of health care professionals with the parents is a key factor in providing support with this process and ensuring that parents feel they are making decisions in an informed manner. In particular, the relationships with healthcare professionals, in this case the midwife, are key to both information gathering and the level of deliberation that is invested in decisions to accept newborn bloodspot screening.
